# Virtual screening following rational drug design based approach for introducing new anti amyloid beta aggregation agent

**DOI:** 10.6026/97320630013042

**Published:** 2017-02-28

**Authors:** Garshasb Rigi, Mohammad Vala Ashdar Nakhaei, Hoda Eidipour, Arshia Najimi, Fahimeh Tajik, Niloufar Taher, Kamran Yarahmadi

**Affiliations:** 1 Department of Biology, Faculty of Science, Behbahan Khatam Alanbia University of Technology, Behbahan, Iran;; 2 Viravigene research institute, Tehran, Iran;

**Keywords:** Alzheimer, rational drug design, Amyloid β, Ligand, docking

## Abstract

Amyloid β (Aβ) sheets aggregations is the main reason of Alzheimer disease. The interacting areas between monomers are residue number 38 to 42. Inhibition of interaction between Aβ molecules prevents plaque formation. In the present study, we have performed a high-throughput virtual screening among ZINC database and top 1000 hits were checked again regarding binding affinity by AutoDock software. Top 4 successive second step screening hits was considered for drug design purpose against aggregation site of Aβ molecules. The toxicity and pharmacological properties of new designed ligands was assessed by PROTOX and FAFdrugs3 webservers. Several steps of modifications performed in the structures of hit#1 and hit#2 and finally new designed ligand based on hit 1, 1-RD-3 (3-[(Z)-6-Hydroxy-4-{[5-(2-methoxyethyl)-6-methyltetrahydro-2H-pyran-2-yl]methyl}-1-methyl-3-hexenyloxy]tetrahydro-2Hpyran- 4-ol) and a designed ligand based on hit 2, 2-RD-2 (6-(Hydroxymethyl)-4-{5-hydroxy-6-methyl-4-[(3- methylcyclohexyl)methyl]tetrahydro-2H-pyran-2-yloxy}tetrahydro-2H-pyran-2,3,5-triol) could successfully pass pharmacological filters. The LD50 of 37000 mg/kg for 1-RD-3 and 2000 mg/kg for 2-RD-2 indicates that the designed ligands can be considered as new candidates for anti Aβ aggregation to treat Alzheimer’s disease. Interestingly, after performing several modification steps still a considerable binding affinity of -9.3 kcal/mol for 1-RD-3 and -9.8 kcal/mol for 2-RD-2 still remained. Theoretically, the new designed molecules can reduce the deposition of Aβ in the cerebral cortex and as the results the Alzheimer symptoms could be decreased.

## Background

Alzheimer's disease (AD) was first described by the German psychiatrist, Alois Alzheimer, in the early 1900s and is now considered the most prevalent progressive neurodegenerative disorder [1]. It causes mental and cognitive deficits such as impaired memory, intellect and personality disorder in people older than 65 years of age. From a histological viewpoint, the progression of AD is associated with 3 cardinal neuro-pathological features: the accumulation of extracellular senile plaques which is mediated by amyloid-beta (Aβ), intracellular neurofibrillary tangles (NFT) and synaptic degeneration. The Aβ peptide is an important risk factor and has a central role in the onset and progression of AD. Soluble Aβ oligomers and their potent neurotoxicity were discovered over a decade ago [1]. ADDLs (nonfibrillar ligands derived from Aβ 1-42) are highly ordered aggregates of amyloid beta 1-42 peptides containing certain multiples of monomer such as trimmers, tetramers, or 12-mers. By contrast, fibrillic amyloid aggregates are amorphous and heterogeneous. ADDLs are soluble, not insoluble like febrile amyloid. ADDLs are ligands, meaning they bind to a specific subset of post-synaptic proteins. ADDL levels are elevated up to 70-fold in Alzheimer’s disease brain tissue compared with agematched control tissue, and they are similarly elevated in Alzheimer’s disease cerebral spinal fluid [2]. It has been shown that the injection of soluble Aβ oligomers into wild-type rat ventricles rapidly compromises execution of an alternating lever cognition model, with complete recovery within 24 hours. Involvement of soluble Aβ oligomers in synaptic malfunction and Alzheimer’s disease associated memory impairment is now widely accepted. Aβ denotes to peptides of 36–43 amino acid which are involved in Alzheimer's disease as the main component of the amyloid plaques. The peptides are primary products of amyloid precursor protein (APP), which is being cut by certain enzymes to yield Aβ [3].

Aβ begins life as a solitary molecule but tends to bunch up initially into small clusters that are still soluble and can travel freely in the brain, and finally into the plaques that are hallmarks of Alzheimer’s. Plaques form when Aβs attaches each other. The most damaging form of Aβ may be groups of a few pieces rather than the plaques themselves [4]. The small clumps may block cellto- cell signaling at synapses. They may also activate immune system cells that trigger inflammation and devour disabled cells. Plaques and tangles tend to spread through the cortex in a predictable pattern as Alzheimer's disease progresses [5]. Since our core expertise is finding the inhibitors and their pharmaceutical model [6,7], in the present study, we tried to use simulation tools in simulated biological conditions to design new chemicals which can be used as a new anti-amyloid beta aggregation agent in a manner that the side effect and un-specific binding of rationally designed molecules reduced.

## Methodology

### Structures

Initially we obtained the crystal structure of Amyloid beta from protein data bank (www.rcsb.org) with PDB number: 1IYT. The structure is a 10 mer aggregated form of amyloid beta. One unit was extracted from the structure and for founding its potential target binding sites; we have submitted the macromolecule to FindSite web server (www.cssb.biology.gatech.edu/finalsite) [8]. After identification of pockets, Zinc database was docked against the pockets and top 1000 ligands was extracted.

### Ligand screening

The monomer structure was added into a cubic water box in the presence of neutralizing ions. The system was then minimized regarding energy level by GROMACS 4.5.6 simulation package [9]. The solved structure was extracted and used as the drug target. Since the most important aggregation site of amyloid plaques are residue number 38 to 42, we defined the second step docking radius in a manner that covers interacting area entirely. The virtual screening process was carried out by PyRx software [10]. PyRx includes Autodock vina with the Lamarckian genetic as scoring algorithm.

### Analysis of pharmacological properties

Top successive hits were then analyzed regarding pharmacological properties by FAF Drugs 3 server and also the un-specific bindings were measured by PROTOX web server [11]. Every single structure passed several modification steps by changing substitutions using HyperChem software [12].

## Result and discussion

In previous studies numbers of isaindigotone derivatives were investigated and some compounds were found to inhibit the acetylcholinesterase and amyloid beta aggregation [13,14]. Several bioinformatics amylases were also studied to introduce the residues may play the role in the analysis of Aβ-peptide by breaking peptide bond [15] and matherials with covered binding affinity requires knowledge about several binding sites in the amyloid β fibril [16].

The most damaging form of Aβ may be groups of a few pieces rather than the plaques themselves [4]. In this study we tried to use simulation tools in simulated biological conditions to design new chemicals which can be used as a new anti-amyloid beta aggregation agent in a manner that the side effect and un-specific binding of rationally designed molecules reduced.

Top 10 successive hits of virtual screening which could gain the most negative binding affinity (-11.2 for hit 1, -11 for hit 2 and - 10.9, -10.9, -10.7, -10.4, -10.1, -9.8, -9.3, -8.7 for hit 3-10 respectively) was considered for further pharmacological analysis. Interestingly, the most appropriate pharmacological features was belonging to hit 1-4. So for the further study we selected them and performed several steps of modifications on them. The results of overall pharmacological features of hit 1-4 are illustrated in table 1 briefly. In the first step of rational drug design, we have checked the hit 1 with FAF Drugs3 and the results indicated that there are several features in the base structure which needs modifications and substitutions to make it a potential drug like compound. Initially there was a problem regarding the molecular weight, log P, number of hetero atoms, number of hydrogen donors and number of hydrogen acceptors. To make this structure more appropriate, we have removed all of the hydroxyl and oxy groups on the surface to decrease number of hydrogen donor and acceptors. In other hands, two rings were removed from the structure to decrease molecular weight. The new ligand, 1-RD-1 (1-(4-Hydroxy-tetrahydro-pyran-3-yloxy)-4-(5-methoxymethoxy- 6-methyl-tetrahydro-pyran-2-ylamino)-hexane-1,6-diol) were then checked by FAF Drugs. The Hydrogen bond related errors and molecular weight problem was solved but the new errors of low risk hemiketal and rotatable bonds appeared. To solve the rotatable bonds problem, we have omitted two lateral methyl groups and one oxygen were replaced by nitrogen. As the results, two single bonds changed to double. Also the hemiketal error was removed by replacing a surface hydroxyl group by methyl. The new rationally designed chemical, 1-RD-2 (N-{6-[1-(2-Hydroxyethyl)- 4-(4-hydroxy-tetrahydro-pyran-3-yloxy)-pent-1- enylamino]-2-methyl-tetrahydro-pyran-3-yl}-formimidic acid methyl ester) was then checked again and the problems regarding hemiketal and rotatable bonds were solved but the modifications in the structure caused new problems such as: low risk enamine and high risk immine. To solve the new problems, two nitrogen were removed. The new modified ligand, 1-RD-3 (3-{6-Hydroxy-4- [5-(2-methoxy-ethyl)-6-methyl-tetrahydro-pyran-2-ylmethyl]-1- methyl-hex-3-enyloxy}-tetrahydro-pyran-4-ol) was checked again and completely accepted in FAF Drugs. Hit 2 (4-[2-Hydroxy-1-(2- hydroxy-1-hydroxymethyl-ethoxy)-3-(3,4,5-trihydroxy-6- hydroxymethyl-tetrahydro-pyran-2-yloxy)-propoxy]-6- hydroxymethyl-6-methyl-tetrahydro-pyran-2,3,5-triol), 3 (2-{4,5- Dihydroxy-2-hydroxymethyl-6-[3-hydroxy-2-(1,2,3-trihydroxypropoxy)- propoxy]-tetrahydro-pyran-3-yloxy}-6-hydroxymethyltetrahydro- pyran-3,4,5-triol) and 4 (6-[3,4,5-Trihydroxy-6-(3,4,5- trihydroxy-6-hydroxymethyl-tetrahydro-pyran-2-yloxymethyl)- tetrahydro-pyran-2-yloxymethyl]-tetrahydro-pyran-2,3,4,5-tetraol) are Laminaran derivate with different substitution positions. Laminaran is known as an anti-coagulant agent and prevents clotting. It also used as the agent for decreasing the lipid level of blood. Laminaran is currently being used to treat hyperlipidemia. Because the basement of the structure of hit 1-4 is very similar, we just focused on of the structures which indicated more negative binding affinity (hit#2). The FAF Drugs results for hit 2 indicated that there are several issues in the structure that needs to be addressed before considering it as a drug like agent. The issues were existence of high risk crown, log p, number of hetero atoms, hydrogen bond donors and acceptors and error in topological polar surface area. In the first step of modification, we have removed 7 hydroxyl group to solve number of hydrogen interactions problem. The new rationally designed ligand 2-RD-1 (6-Hydroxymethyl-4-[5-hydroxy-6-methyl-4-(6-methyltetrahydro- pyran-2-yloxy)-tetrahydro-pyran-2-yloxy]-tetrahydropyran- 2,3,5-triol) checked again and the errors related to hydrogen bond donor and acceptors, log p, topological polar surface area and number of hetero atoms was solved. But still the error related to high risk crown was still exist. Finally we designed 2-RD-2 and in the new structure we have replaced two oxygen with carbon. One in the ring and the other in the linker between rings have been replaced to reach a new structure (6-Hydroxymethyl-4-[5- hydroxy-6-methyl-4-(3-methyl-cyclohexylmethyl)-tetrahydropyran- 2-yloxy]-tetrahydro-pyran-2,3,5-triol) which could be accepted as a drug like in FAF Drugs3 web server. For supplementary analysis, we have checked the new rationally designed ligands (1-RD-3 and 2-RD-2) for oral toxicity prediction. For gaining this purpose, we predicted the toxicity of our designed ligands based on chemical similarities between compounds with known toxic effects and the presence of toxic fragments. The PROTOX results indicated that 1-RD-3 has a predicted LD50 of 37000 mg/kg with the toxicity class of 6 (1most toxic and 6 less toxic). Also two possible toxicity targets were predicted for 1-RD-3. Its pharmacophore was predicted to have the similarity of 39.1 % fit with Amine Oxidase A and 37.23% similarity fit with Prostaglandin G/H Synthase 1. Bus because the predicted LD50 indicates very low toxicity value and in other hand, the average pharmacophore fit is not considerable, it can be deducted that the 1-RD-3 theoretically is an anti- A beta aggregation agent. Regarding 2-RD-2, the predicted LD50 was 2000 mg/kg which indicated the toxicity class of 4. Interestingly, no un-specific target binding was identified for this chemical. The predicted toxicity level was due to similarity with known toxic chemicals.

## Conclusion

The rationally designed ligands (1-RD-3 and 2-RD-2) has passed several modification steps but their binding affinity was still in a level which can be claimed that they can interact properly with Aβs molecules and preventing them from forming plaques and depositing in the cerebral cortex. In other hands, the LD50 value of rationally designed ligands indicates that 1-RD-3 and 2-RD-2 have low toxicity values. Theoretically, the new designed molecules can reduce the deposition of Aβ in the cerebral cortex and as the results the Alzheimer clinical symptoms will be decreased in the presence designed ligands.

## Figures and Tables

**Table 1:** The pharmacological properties of top 4 hit and the rationally designed ligands.

Hit#	MW	LogP	Rotatable bonds	Flexibility	HBD	HBA	Ring	Solubility (mg/l)	Binding affinity
1	666.58	-9.02	10	0.29	14	21	4	712934.801	-11.2
2	504.44	-5.78	7	0.28	11	16	3	157078.884	-11
3	504.44	-6.88	7	0.28	11	16	3	314111.534	-10.9
4	504.44	-6.88	7	0.28	11	16	3	314111.534	-10.9
1-RD-1	396.41	0.37	12	0.5	4	9	2	69658.24	-9.6
1-RD-2	391.44	1.81	10	0.42	3	8	2	25093.55	-9.7
1-RD-3	391.48	2.32	11	0.46	2	6	2	19436.76	-9.3
2-RD-1	402.39	-0.88	5	0.22	5	10	3	94348.23	-10.1
2-RD-2	398.45	1.52	5	0.22	5	10	3	21106.93	-9.8

**Figure 1:**
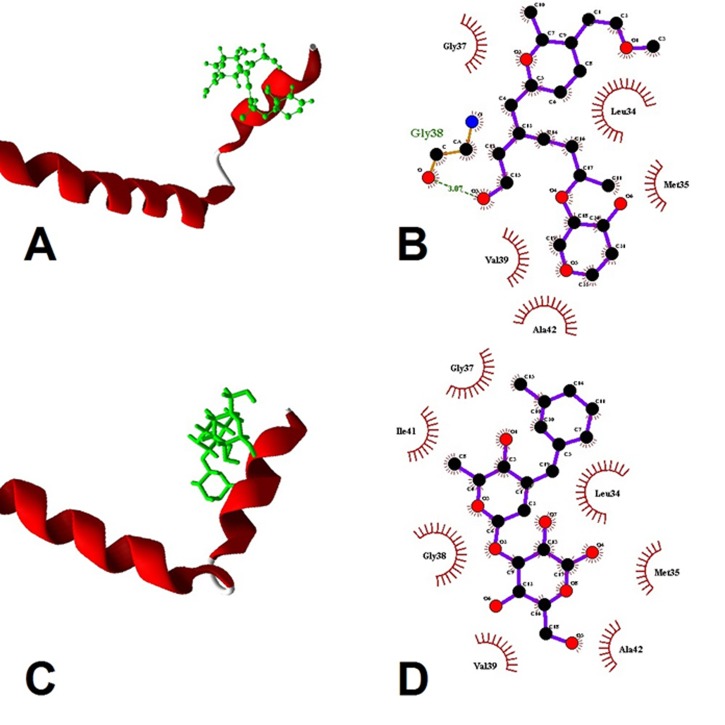
Molecular illustration of target protein amyloid beta with docked compounds. (A) 1-RD-3 with target monomer; (B) ligand map of 1-RD-3 with target monomer; It interacts with: Gly 37, Leu34, Val 39, Met 35, Ala 42. Binding affinity: -9.3 Kcal/mol. (C) 2-RD-2 with target monomer; (D) ligand map of 2-RD-2 with target monomer. It interacts with Leu 34, Met 35, Gly 37, Gly 38, Val 39, Ile 41 and Ala 42. Binding affinity: -9.8 Kcal/mol.
